# Cross sectional associations of screen time and outdoor play with social skills in preschool children

**DOI:** 10.1371/journal.pone.0193700

**Published:** 2018-04-04

**Authors:** Trina Hinkley, Helen Brown, Valerie Carson, Megan Teychenne

**Affiliations:** 1 Deakin University, Institute for Physical Activity and Nutrition, Burwood, Victoria, Australia; 2 Faculty of Physical Education and Recreation, University of Alberta, Edmonton, Alberta, Canada; IRCCS E. Medea, ITALY

## Abstract

Screen time and physical activity behaviours develop during the crucial early childhood period (0–5 years) and impact multiple health and developmental outcomes, including psychosocial wellbeing. Social skills, one component of psychosocial wellbeing, are vital for children’s school readiness and future mental health. This study investigates potential associations of screen time and outdoor play (as a proxy for physical activity) with social skills. Cross sectional data were available for 575 mothers with a child (54% boys) aged 2–5 years. Mothers reported their child’s screen time, outdoor play time and social skills (Adaptive Social Behavior Inventory; ASBI). Multiple linear regression analyses assessed associations of screen and outdoor play time (Model 1) and compliance with screen time and physical activity recommendations (Model 2) with three ASBI subscales. Boys and girls spent a mean of 2.0 and 2.2 hours per day in screen time, and 3.3 and 2.9 hours per day in outdoor play, respectively. Girls scores for express and comply skills were significantly higher than boys (p<0.005). After applying the Benjamini-Hochberg Procedure to adjust for multiple associations, children’s television/DVD/video viewing was inversely associated with their compliant scores (B = -0.35 95% CI -0.26, -0.14; p = 0.001) and outdoor play time was positively associated with both expressive (B = 0.20 95% CI 0.07, 0.34; p = 0.004) and compliant (B = 0.22 95% CI 0.08, 0.36; p = 0.002) scores. Findings indicate that television/DVD/video viewing may be adversely, and outdoor play favourably, associated with preschool children’s social skills. Future research is required to identify the direction of causation and explore potential mechanisms of association.

## Introduction

Screen time (i.e. time spent viewing or use of a device with a screen, including, but not limited to television, DVDs, electronic games and computers [1]) and physical activity are two health-related risk behaviours, which typically develop during the crucial early childhood period [2, 3]. Once established, these behaviours tend to track, or remain stable, into middle childhood [4]. These behaviours are also associated with important health and developmental outcomes, such as weight, bone health, cardiovascular disease risk factors [5, 6], cognitive development, academic achievement [7–9], and psychosocial well-being [10–12] from early childhood through to adolescence. That is, higher levels of physical activity and lower levels of screen time are supportive of more favourable outcomes.

Positive indicators of children’s psychosocial well-being during early and middle childhood have been shown to be inversely associated with later depression, hostile behaviour and aggressive interpersonal behaviour [13–15]. Social skills (e.g. openness of expression, cooperation) are intrinsic [16, 17] to children’s psychosocial well-being. These skills may also support children’s positive behavioural, social and academic outcomes during later childhood [18, 19]. Specifically, young children’s social skills are an important precursor in their development of school readiness [20] and peer acceptance [21]. Therefore, ensuring preschoolers have sufficient opportunities to develop and practice their social skills may establish a foundation for their long-term health and well-being.

Previous studies have suggested that participation in play and physical activity may provide one way of supporting the development of children’s social skills [22, 23] as these behaviours offer opportunities for interaction [24] where children may observe and practice such skills. Outdoor playtime has previously been shown to be consistently associated with physical activity, and inversely associated with sedentary time, across studies in preschoolers such that children who spend more time outside are more active and less sedentary than children who spend less time outside [25–28]. Outdoor playtime has been validated [29] and is often used as a proxy measure [30–33] of physical activity in this population, including as a means of assessing children’s compliance with physical activity recommendations [33]. Further, outdoor play has been shown to facilitate interaction with peers, thereby providing more opportunities to support socialisation and the development of social skills [34–36] and children’s overall social health [37, 38]. Therefore, outdoor play provides opportunities for children to be both physically active and socially engaged.

Conversely, participation in screen based behaviours, which are largely solitary, sedentary pursuits, may reduce opportunities for interaction with peers and thereby be detrimental to children’s opportunities for socialisation and to development of healthy social skills [39, 40]. Concern has also been raised about the impact of screen time given its potential to reduce time in which children may engage in developmentally appropriate activities [41] and it has recently been reported as parents’ number one concern for their children [42]. Additionally, television viewing has been shown to be associated with delays in cognitive, language and motor development in young children [43]. These skills are necessary to support children’s social interactions with peers and adults and, if compromised, may subsequently affect children’s opportunities for positive social interactions. Therefore, it is imperative to explore potential associations between these behaviours and preschoolers’ social skills.

No previous studies have investigated associations of physical activity with social skills [17] during the preschool period (roughly two to six years of age [44, 45]) and only three studies have investigated associations of television viewing and social skills [17] in that age group. However, findings from those studies are conflicting, with positive, inverse and null associations between television viewing and social skills reported across studies [46–48]. No studies have investigated associations of other types of screen time with social skills in preschool children. As such, there is a need to further explore the potential association of television viewing, other types of screen time, and outdoor play with preschoolers’ social skills. Given reported differences in boys’ and girls’ physical activity [49] and social skills [50], and differences in children’s socialisation by sex [51, 52], it may be that children’s physical activity and screen time behaviours, social skills and the associations between those variables also vary by sex. However, no previous research has investigated such a possibility. This study therefore sought to investigate associations of multiple indicators of screen time and outdoor playtime with social skills in preschool children (defined herein as 2–5 years of age) and to determine if differences varied by child sex. Hypothesised findings were that screen time would be adversely, and outdoor play time favourably, associated with preschool children’s social skills.

## Methods

### Study sample

The ‘Mums, Dads and Kids activity and screen time study’ (MDK) was a cross-sectional, online study where parents of a child aged two to five years (who were not yet in formal schooling) were recruited between September 2013 and March 2014 (early spring to early autumn). MDK obtained ethical approval from Deakin University, Faculty of Health, Human Ethics Advisory Group (HEAG-H 138_2012) and the Department of Education, Employment and Childhood Development. Reporting follows the STROBE recommendations [53, 54].

Participants were recruited through preschools, childcare centres and early childhood activity groups (e.g. music or gym groups) in six randomly selected, socioeconomically diverse [55] local government areas (LGA’s) within metropolitan Melbourne and through online advertising via blogs and Facebook pages related to parenting, child education and family-lifestyle. In total, 191 LGA-based facilities were contacted. Fifty-nine preschools/childcare centres (30.9%) and 81 early childhood activity groups (42.4%) agreed to distribute information regarding the study to parents. A total of 30 Facebook profile administrators and 36 online blog authors were contacted: 15 Facebook profile administrators (50%) and 10 online blog administrators (27.8%) agreed to post study information. Participants were directed to a website where they were screened to ensure eligibility for participation (inclusion: at least one child aged 2–5 years who had not yet started school) and provided informed consent prior to completing an online survey.

A total of 1238 parents were screened; of those, 958 were eligible and were provided with personalized links to complete a mother’s or a father’s survey: the respondent parent was asked to complete the survey relevant to them (either the mother’s or the father’s survey) and to forward the link for the other survey to their partner/spouse. Due to the overall objectives of the project, only the mother’s survey assessed children’s social skills. A total of 679 participants commenced the mother’s survey. From that dataset, eight participants were excluded due to duplicate identification numbers where one version was only partially completed; 13 participants were excluded due to the child age being outside the age range and researchers being unable to obtain clarification; one participant was excluded due to the respondent being male; and two participants were excluded where maternal date of birth was invalid (year of reporting rather than birth year) and respondents were unable to be contacted. In total, 655 (96.5%) cases were available. However, 80 cases had missing data and full data were available for 575 (84.7%) families: 311 boys (54%) and 264 girls. All analyses included the full sample of 575 participants with complete data.

### Measures and data management

#### Dependent variables

Each mother reported on their child’s social skills using the Adaptive Social Behaviour Inventory (ASBI) [56, 57]. The ASBI is a reliable [56, 57], 30 item inventory which assesses children’s social skills across three subscales: Express (e.g. joins play, is open and direct; 13 items), Comply (e.g. cooperates, is calm and easy going; 10 items) and Disrupt (e.g. teases, bullies; 7 items). These subscales were identified in a previous study through factor analysis from an original pool of 83 items [57] and have been shown to have high levels of internal reliability (α = 0.71–0.87) and generally moderate reliability against the Child Behavior Checklist (r = 0.24–0.63)[56, 57]. Mothers rated their child for each item on a three point Likert scale: rarely/never, sometimes or always. Items were summed for each of the sub-scales. All dependent variables were normally distributed.

#### Independent variables

Mothers reported their child’s time (in hours and minutes) in each of the following behaviors separately for week and weekend days (total of 6 items):

1. television/DVD/video viewing;
2. computer/electronic game/hand held device use; and
3. outdoor play time as a proxy for physical activity.

Mothers were directed to consider only that time when the behaviour in the question was the main activity their child was doing. All screen time items had established test-retest reliability (ICC = 0.68–0.69) [58]; outdoor play items had been previously validated against accelerometry (r = 0.2, p = 0.003) [29]. Data were transformed to average hours per day in each behaviour as follows: [(weekday hours * 5) + (weekend hours * 2)]/7. Average hours per day in total screen time (television/DVD/video viewing and computer/electronic game/hand held device use combined) and outdoor play were dichotomised to determine children’s compliance with Australian recommendations [59] for total screen time of less than one hour per day and physical activity of three or more hours per day. Thus, children were determined to be compliant with the screen time guideline if they spent less than one hour per day in screen time, and were determined to be compliant with the physical activity guideline if they spent three or more hours per day in outdoor play.

#### Covariates

Mothers reported on a variety of their family’s demographic characteristics. For the purposes of this study, mothers reported their own highest level of education used as a proxy indicator of family socioeconomic position (SEP; low SEP: year 10 or less education; mid-SEP: year 12, diploma, trade; high SEP: university or higher qualification), their child’s sex and date of birth (from which child age at the time of survey completion was determined), and whether or not their child had a disability. These variables were chosen as covariates based on previous studies showing that child social skills differ between the sexes (girls tend to perform more strongly), improve with age [60–62], and are poorer in children with a disability [63–65]. Maternal education has also been shown to be a predictor of preschool children’s social skills, including in studies using the ASBI [66–68].

### Statistical analyses

Descriptive statistics were used to describe the sample. T-tests were used to assess differences in independent and dependent variables between boys and girls where data were continuous and Chi square tests were used to assess sex differences for the dichotomous variables, which report compliance with recommendations. Correlations between all the independent variables, and between all the dependent variables, were assessed. Two multiple linear regression models were run for each of the outcome variables:

1. Model 1 included continuous variables for each of the 3 independent variables—television/DVD/video viewing, computer/electronic game/hand held device use and outdoor play time.
2. Model 2 included 2 dichotomised variables for compliance with the screen time and physical activity recommendations as the independent variables.

Both models controlled for the covariates identified above. All models were assessed for an interaction between sex and the independent variables; as there were no significant interactions in any of the models, the interaction term was removed and all analyses were undertaken for the whole sample controlling for sex as a covariate. All analyses were undertaken in Stata SE 13.1 (Statacorp, College Station, Texas). Data were analysed in 2015–2017. Statistical significance was set at p<0.05. All significance values for coefficients of independent variables were assessed against the Benjamini-Hochberg Procedure [69] for controlling for false discovery rates. For the 15 coefficients of independent variables tested, the adjusted p-value was determined to be 0.01; therefore, coefficients of independent variables had to be significant at p<0.01 after adjustment to maintain significance.

## Results

Boys and girls had a mean age of 3.8 (1.0) and 3.7 (0.9) years, respectively, and 8.5% of the sample (11.1% of boys and 5.4% of girls, *p* = 0.012) had a parent-reported disability. Three percent of the sample were classified as low SEP based on maternal education; 22% were mid-SEP and 75% were high SEP.

Table 1 reports scores for each of the ASBI subscales and overall social skills for boys, girls and the whole sample. Girls’ scores for the expressive (mean = 35.8, 95%CI 35.4, 36.1) and comply (mean = 25.5, 95%CI 25.2, 25.9) subscales, and for overall social skills (mean = 77.2, 95%CI76.6, 77.9), were significantly higher than boys’ scores (35.0, 95%CI 34.6, 35.4; 24.8, 95%CI 24.4, 25.2; 75.8, 95%CI 75.0, 76.6, respectively). No difference was seen between girls’ and boys’ scores on the disrupt subscale.

**Table 1:** Screen time, outdoor play and social skills characteristics for boys, girls and the whole sample from the 2013/2014 MDK participants.

	Boys (n = 311)		Girls (n = 264)		All children (n = 575)	
	mean	95%CI	mean	95%CI	mean	95%CI
**ASBI social skills**						
Expressive	**35.0**	34.6, 35.4**	35.8	35.4, 36.1	35.4	35.1, 35.6
Comply	**24.8**	24.4, 25.2**	25.5	25.2, 25.9	25.1	24.9, 25.4
Disrupt	10.3	10.1, 10.4	10.2	10.0, 10.4	10.2	10.1, 10.4
Overall social skills	**75.8**	75.0, 76.6**	77.2	76.6, 77.9	76.5	75.9, 77.0
**Screen time and outdoor play variables (hours/day)**						
Average daily screen time	2.03	1.85, 2.00	2.19	1.96, 2.42	2.1	1.96, 2.2
Average daily television/DVD/video viewing	1.45	1.32, 1.58	1.67	1.49, 1.85	1.6	1.4, 1.7
Average daily computer/e-game/hand held game use	0.58	0.49, 0.66	0.52	0.43, 0.61	0.5	0.5, 0.6
Average daily outdoor play	3.26	3.04, 3.48	2.94	2.71, 3.17	3.1	3.0, 3.3
Meeting daily screen time recommendations (% compliant)	27.5	-	27.6	-	27.6	-
Meeting daily physical activity recommendations based on outdoor play (% compliant)	45.3	-	35.8*	-	40.9	-

Boldface indicates statistical significance (* *p*<0.05, ** *p*<0.01).

Table 1 shows the mean daily hours in the screen time variables and outdoor play for boys, girls and the whole sample. Nearly 20% more boys met the physical activity recommendations than girls (45.3% vs. 35.8%). There were no other significant between-sex differences in any of the screen time variables or outdoor play.

Correlations between all dependent variables are shown in Table 2 and those for independent variables are shown in Table 3. Express and comply social skills scores were moderately positively and significantly correlated while disrupt was weakly and inversely significantly correlated with both express and comply scores. Independent variables were all weakly, significantly and positively correlated.

**Table 2:** Correlations for all dependent variables.

	Express *r* (p)	Comply *r* (p)	Disrupt *r* (p)
Express	-		
Comply	0.53 (<0.001)	-	
Disrupt	-0.09 (0.03)	-0.34 (<0.001)	-

**Table 3:** Correlations for all independent variables.

	TV *r* (p)	Computer *r* (p)	Outdoor play *r* (p)
TV	-		
Computer	0.34 (<0.001)	-	
Outdoor play	0.28 (<0.001)	0.19 (<0.001)	

Table 4 reports the results of the regression models where television/DVD/video viewing, computer/e-game/hand held game use and outdoor play time (Model 1), or meeting screen time and physical activity recommendations (Model 2), were entered simultaneously as independent variables. Following adjustment using the Benjamini-Hochberg procedure, two significant associations were evident. These included:

1. children’s television/DVD/video viewing was inversely associated with their compliant scores (B = -0.35 95% CI -0.26, -0.14; p = 0.001) and
2. outdoor play time was positively associated with both expressive (B = 0.20 95% CI 0.07, 0.34; p = 0.004) and compliant (B = 0.22 95% CI 0.08, 0.36; p = 0.002) scores.

**Table 4:** Associations from multiple linear regression models of ASBI sub scales with screen time and outdoor play (hours/day) entered simultaneously for 2013/2014 MDK participants (coefficients, 95%CI; p values).

	Expressive subscale	Compliant subscale	Disrupt subscale
**Model 1: Average daily time (hours/day)**			
*Independent variables*			
Average daily television/DVD/ video viewing	-0.23 (-0.43, -0.02; 0.034)	**-0.35 (-0.26, -0.14; 0.001)**	0.04 (-0.09, 0.16; 0.549)
Computer	-0.12 (-0.53, 0.28; 0.551)	-0.25 (-0.66, 0.16; 0.240)	0.14 (-0.11, 0.38; 0.276)
Average daily outdoor play	**0.20 (0.07, 0.34; 0.004)**	**0.22 (0.08, 0.36; 0.002)**	-0.10 (-0.19, -0.02; 0.015)
*Covariates*			
Child age	0.79 (0.52, 1.06; <0.001)	1.12 (0.84, 1.39; <0.001)	0.05 (-0.12, 0.21; 0.573)
Family SEP	-0.19 (-0.69, 0.32; 0.469)	0.08 (-0.43, 0.59; 0.759)	-0.03 (-0.33, 0.27; 0.842)
Child disability (ref: yes)	2.42 (1.51, 3.32; <0.001)	1.84 (0.92, 2.77; <0.001)	0.28 (-0.26, 0.83; 0.310)
Child sex (ref: male)	0.79 (0.27, 1.30; 0.003)	0.87 (0.35, 1.40; 0.001)	-0.10 (-0.41, 0.21; 0.518)
**Model 2: Meeting recommendations**			
Meeting screen time recommendations (ref: no)	0.17 (-0.42, 0.75; 0.573)	0.65 (0.05, 1.25; 0.033)	-0.32 (-0.67, 0.03; 0.072)
Meeting physical activity recommendations based on outdoor play (ref: no)	0.47 (-0.05, 0.99; 0.079)	0.26 (-0.28, 0.79; 0.346)	-0.13 (-0.44, 0.19; 0.432)
*Covariates*			
Child age (years)	0.76 (0.48, 1.04; <0.001)	1.11 (0.83, 1.39; <0.001)	0.03 (-0.13, 0.20; 0.713)
Family SEP	-0.13 (-0.63, 0.37; 0.616)	0.15 (-0.36, 0.66; 0.563)	-0.01 (-0.31, 0.29; 0.971)
Child disability (ref: yes)	2.43 (1.52, 3.35; <0.001)	1.81 (0.87, 2.74; <0.001)	0.31 (-0.24, 0.86; 0.263)
Child sex (ref: male)	0.73 (0.21, 1.25; 0.006)	0.77 (0.25, 1.30; 0.004)	-0.08 (-0.39, 0.23; 0.600)

Boldface for independent variables indicates statistical significance after applying the Benjamini-Hochberg adjustment for false discovery rates where p<0.01 was the cut-off for significance of coefficients of independent variables.

## Discussion

This paper sought to investigate associations of multiple indicators of screen time and outdoor play with social skills in preschool children. These findings suggest that children’s higher levels of screen time, and lower levels of outdoor play, are associated with poorer social skills, particularly children’s compliant skills. Overall, coefficients of association were small; however, consistency between independent variables for children’s compliant skills, and across dependent variables for children’s outdoor play, suggests that these associations may have practical significance.

This study found differences in associations between television/DVD/video viewing and computer/e-game/hand held game use with children’s social skills. Television/DVD/video viewing was inversely associated with compliant skills while computer/e-game/hand held game use showed no association with any of the social skills scales. This finding is consistent with previous studies which similarly report inverse associations between TV viewing and prosocial behaviour [70], cooperation, assertion, self-control and overall social skills [47]. Although few studies have investigated associations between various types of screen devices and indicators of children’s psychosocial well-being such as social skills, those that have done so show different associations between screen time devices and well-being indicators. For instance, television/video viewing has been shown to be associated with emotional well-being, while computer use was associated with emotional problems in the same sample [10]. This is not surprising given the type of content, interaction, behaviour and engagement differs between devices. For instance, during television/DVD/video viewing, children are more likely to be passive consumers of content, while computer, electronic game and smart phone/digital tablet use may elicit more interactive behaviours and engagement [41] between child and content engaged with as well as between children and parents during device use. Such differences may be due to differences in content (e.g. television cartoons or drama programs vs. games which require children to respond or video chatting) or opportunities for interaction with others during use (e.g. video chatting, multi-player games). Further, game playing of all types, including outdoor and sedentary games, is a normal part of childhood and moderate levels of computer game playing have been shown to be associated with favourable outcomes (e.g. activity involvement, positive school engagement, positive mental health, self-concept, friendship network, and low levels of disobedience to parents) in adolescents [71]. No such associations were found in this study, possibly due to the small amount of time (approx. 30 mins/day) children spend in combined computer use and game playing compared to the amount of time (2 h 44 min) adolescents spend in such pastimes [72]. Given such differences, it is essential that future research studies ensure they collect data on a range of screen devices, such as television, computer, and digital tablets, as well as type of content and interaction during use, to more comprehensively examine the components of screen time, which may support or constrain favourable outcomes for children.

This study found that girls had significantly higher scores on the expressive and compliant subscales than boys. Girls have previously been reported to have higher levels of interpersonal and intrapersonal skills [50], which may be similar to the social skills constructs captured in this study. It is possible that characteristics of the socialisation process may account for these differences [51, 52, 73]. Mothers are reported to be more consistently emotionally expressive than fathers [74, 75], and express more positive emotions with their daughters than their sons [76], thereby modelling such behaviours to their children. Parents also reportedly desire differences in emotional development in their sons compared with their daughters. For instance, parents desire more anger suppression in their sons than their daughters [77]. Given that experience and expression of emotion have been shown to be socially constructed rather than innate [78, 79], it is possible that differences in girls’ and boys’ social skills scores in this study are a result of socialisation. However, it is beyond the scope of this study to test this assumption in the current sample.

Evidence is not clear on the association of sedentary behaviors, such as screen time, or physical activity, in children with health or developmental outcomes independently of each other [80]. However, evidence in adults suggests that these behaviors may be independently associated with mental health outcomes [81–83].The results of Model 1, which includes individual screen and outdoor play time variables, are consistent with those findings in the adult literature such that associations of television viewing and outdoor playtime with children’s social skills may be independent of each other. That is, even children who spend a large amount of time playing outdoors may suffer adverse effects of higher volumes of television viewing, and vice versa. Given the relatively low prevalence of compliance with recommendations, and the potential associations with health and psychosocial well-being, it is essential that future research further investigates, and implements programs to support healthy levels of these behaviors during early childhood.

There is currently no evidence to identify what possible causal mechanisms may exist between preschool children’s screen time, outdoor play (as a proxy for physical activity) and their social skills. However, some hypotheses may be useful. As play and physical activity provide opportunities for interaction [24], specifically conversation, cooperation and management of conflict, children may learn important social skills through their everyday play and activities. Conversely, participation in screen time, especially passive forms, may limit children’s opportunities to interact with others. Indeed, parent-child interaction has been shown to decrease in the presence of background television [84]. The social withdrawal hypothesis, suggested as a possible causal mechanism between increased television viewing and depression in adults [39, 40], may have some applicability in explaining the associations identified in this study also. That hypothesis suggests that as individual engage in solitary behaviours, such as television viewing, they are withdrawn from social behaviours which may support aspects of wellbeing and mental health. Alternatively, television viewing has been found to be inversely associated with sleep in children, both cross-sectionally during the preschool period and longitudinally from early childhood into middle childhood [85–88]. Therefore, children who watch more television, may sleep less, or have poorer quality sleep, which may subsequently reduce their ability to be calm, disrupting their capacity to be compliant and behave appropriately in social situations. Nonetheless, it remains for future research to investigate such potential causal mechanisms.

### Strengths and limitations

Strengths and limitations of the study must be acknowledged. The cross-sectional design precludes identification of causality: indeed, it may be that social skills predict screen time and physical activity. The sample was highly educated with 75% of mothers having completed a university degree or higher. Therefore, it is possible that these findings may not be generalizable to families where mothers are less educated. Nonetheless, statistical models included maternal education as an indicator of SEP to control for the potential confounding influence of this characteristic. The findings also may not be generalizable to families of different combinations, such as single-father or grandparent-headed families. Nonetheless, 81% of families in Australia with a child aged 0–4 years are two parent families, and 16% of families are lone mother families; thus these findings are likely to be characteristic of the majority of children [89].

Although this study assessed a range of child screen time behaviours, capturing participation in more recent screen devices such as hand held devices, this time was reported across multiple, rather than individual, devices and therefore associations between use of individual devices and social skills were not able to be ascertained. Further, although it is likely that mothers would have included their child’s use of smart phones and digital tablets in their response to this item, the survey item did not specifically prompt for inclusion of use of those devices. As levels and types of engagement and participation vary between devices [41], it is possible that associations do also. Nonetheless, this is one of the first studies to include investigation of the association of such modern devices with children’s social skills, where most previous studies have primarily captured only television/DVD/video viewing.

The study used only proxy-report of the child’s time in each of the independent variables: with the exception of behavioural observation which is highly resource-intensive, no objective measures of screen time are currently available and it was not feasible to use accelerometry in this study. Children’s outdoor play has been weakly correlated with accelerometry [29], is consistently associated with children’s levels of physical activity across studies [49], and has previously been used as an indicator of children’s compliance with guidelines [33]. However, this measure does not capture all of the children’s physical activity behaviours and neither is all outdoor play time physically active. Further, objective measures may result in different levels of compliance with physical activity recommendations and associations with outcomes being identified.

Children in this study spent a mean of 16.4 (SD = 11.9; median 15) hours per week in childcare/preschool and 77.6% of mothers reported that they are the primary carer for their child, providing 75% or more of the child’s care outside of formal care hours. Therefore, the children in this study spent the majority of their time outside of care with their mothers. It is reasonable that mothers are therefore reporting the majority of children’s outdoor play throughout the week. Proxy-report measures are also subject to biases which, for this study, may include over-reporting of girls’ social skills and boys’ outdoor play.

Data were collected from only one respondent which may add additional bias and precludes triangulation. Nonetheless, evidence from previous studies included above suggests that these perceptions are consistent with previous research and objective physical activity monitoring. As these items were parent-reported, reported time is unlikely to include any time in these behaviours while children were not in the mothers’ care. Therefore, time children participate in screen time while in formal or informal care was not specifically included. However, models were tested with time in childcare as a covariate; only minimal differences in associations (at the second decimal place in the coefficient and p values) were observed and therefore time in childcare was not included in the models. Finally, there may be other factors more strongly associated with children’s social skills than those captured in this study, such as maternal anxiety or depression, which may be worth exploring.

## Conclusion

In summary, this study has shown that television/DVD/video viewing, total screen time and outdoor play are associated with preschool children’s social skills. Future longitudinal studies are required to assess the temporal associations between children’s behaviours and indicators of their well-being to determine causality and use of national datasets, where available, would add substantially to this body of literature. Nonetheless, such findings may aid in the development of interventions to support healthy outcomes in preschool children. Additionally, these findings may suggest a possible stealth approach to interventions is feasible [90]. Research has shown that parents are largely unconcerned about their children’s physical activity and screen time behaviours and report multiple reasons to maintain current behaviours [91]. A stealth intervention, targeting outcomes of value and saliency to parents such as children’s well-being, may be successful in engaging and motivating parents to participate and support healthful behaviour change for their children.
